# 

**Published:** 1817-12

**Authors:** 


### INDEX

### TO THE THIRTY-EIGHTH VOLUME.

Abdomen, case where it was perforated by vermes lumbricus 132
———, on wounds in the 371
Aberdeen, observations on the harbour of 170
Abernethy, Mr. account of his lectures 26
———, on his treatment of lumbar abscesses 59
———, case of the medulla sarcoma described by him 152
——— on pseudo-syphilitic diseases 314, 456
Abscesses of the trachea, cases of 53
———, new method of treating lumbar 59
Abstinence, extraordinary instance of 341
Accum, Frederick, account of his chemical amusements 422
Acid, case of swallowing sulphuric 129
——, on the antisyphilitic virtue of the nitric 429
——, on the use of nitro-muriatic 466
Acton, Mr. on calcareous matter taken from horses 348
Acupuncture, description of that operation 436
Adams' solvent, cure effected by 36
Adams, Dr. his observations on epilepsy 39, 126
———, French translation of his Life of Hunter 77
———, extracts from the same work 107
———, his cases of periodical sickness cured by arsenic 129
——— chosen a member of the Society of Medicine at Paris 260
——— announcement of a new edition of his Life of Hunter 263
———, his memorial on elephantiasis 289
Africa, peculiar propensity to dirt-eating in the negroes of 79
———, account of a remarkable female from 335
Agents, on organic and inorganic 2
Air, effects of the atmospheric 79
Akenside, Dr. anecdotes of 120
Alcester, account of plants growing about 240
Alchemy, on the Arabian origin of 203
Alcohol, process of distilling it from potatoes 432
Alderson, Dr. the priority of his claim to the method of treating wounds of the chest 38
Aldridge, Mr. his account of sulphuric acid swallowed 129
Algiers, report on the wounded in the action before 411
Alibert, M. on chronic eruptions 21
———, review of his Elements of Therapeutics and Materia Medica 330
Alisma plantago, its virtue in hydrophobia 435
America, miscellaneous intelligence from thence 165
Aminicus, his observations on a late trial 95
——— on poison in the stomach 96
Amputation, cautions in regard to 63
———, method of treatment after the operation 66
———, method of practising it in the Tonga Islands 202
———, on the operation of it in wounds and fractures 478
Analysis of new Publications 48, 126, 210, 306, 403, 476
Anatomy, practical observations on morbid 48
———, review of a new system of 414
———, morbid, of the brain in typhus fever 491
Annular saw, improvements in the 348, 516
Anecdotes—of John Hunter, 107; of Dr. Lettsom, 117; of Dr. Akenside, 120; Dr. Valli, 345; Dr. Ingenhousz, 377; of Guyton de Morveau, 382; Dr. W. Saunders, 388; Dr. Combe, 392; Dr. Wells, 395; Professor Werner, 397; Medical fracas, 430, 516.

Aneurism, instance of spontaneous cure of inguinal 60
——, new method of applying ligatures in 68
——, case of femoral, cured by tying the external iliac artery 489
Antelope, yellow fever conveyed by his Majesty's ship 144
Animals, on the powers by which they are connected with the external world 8
—— have their geography 334
Anus, instance of one in the groin 476
Apoplexy, that term too widely used 27
——, characteristics of 494
Apothecaries, meeting of the Association of 174
——, observations on the act for regulating 249
——, eighth report of their General Committee 252
Apprentices, advice to medical 31
——, hardship of those of apothecaries 349
Arabs, on the state of medical knowledge among the ancient 203
——, on the leprosy of the 302
——, elephantiasis common among them 307
Aretæus, his observations on epilepsy 39
—— on elephantiasis 296
Army, hints to practitioners in the 441
Arsenic, observations on its effects in the stomach 92
——, experiments for detecting it 94
——, on a supposed case of poison by 95
——, method of ascertaining its presence in the stomach 96
——, periodical sickness cured by 129
——, on the different tests for the detection of 172
——, remarks on it as a poison, with five cases of recovery 240
——, on an additional test for 252
——, experiments on the poison of 446
Arteries, on the course of the blood through the 10
——, on stopping the bleeding of divided 12
——, on their contractile power ib.
——, proposed experiment on that subject 13
——, on tying the ligature on them ib.
Arteries, on tying the internal iliac one 267
——, on the relation of nerves with them 499
Atkinson, James, on tying the internal iliac artery 267
——, description of an instrument invented by him for that operation 272
——, Thomas, on the symptoms, prognosis, and treatment of internal hæmorrhage after delivery 462
Atmosphere, its effects in changing the human skin 79
——, effects of vitiated 151
——, on its influence in the British isles 437
Babington, Dr. patronised by Dr. Saunders 391
Bagnold, Captain, on the fatal effects ascribed to the wind of a cannon ball 265, 373
Baillie, Dr. extract from his Morbid Anatomy 113
Balfour, Dr. on the vindication of 25
——, his cases of re-union of the toes 250
Balls, on wounds in the chest from 38
——, on the effects ascribed to the wind of 265, 373
Bancroft, Dr. his confused notion of contagion 17
Barbadoes leg, observations on the 301, 308
Bark, improved preparation of 81
Baron, Dr. John, his case of ruptured brain 464
Bateman, Dr. on syphilis 21
——, on his practical synopsis of cutaneous diseases 309, 310
—— on leuce and lepra græcorum 312
——, anecdotes of him 404
Bath, description of the nitro-muriatic acid one 466
Battersea, botanical researches at 85, 86, 349, 437, 438
Bayswater, plants growing there 347
Beauvois, M. account of his discoveries in natural history 333
Beclard, M. on a case of strangulated intestines 170
——, his case of reversed vagina 342
Bees, said to have been found in a block of stone 519
Bell, Charles, his Surgical Observations reviewed, with extracts 476
—— on ligatures 13
——, John, on the pressure of tumours 479

Berlioz, Dr. on evacuations and acupuncture in chronic disease 436
Bichat, M. observations on his physiology 9
——, character of him 416
Biography—of John Hunter, 107; of Dr. Lettsom, 117; Madame de Stael, 293; Dr. Valli, 345; Dr. Ingenhousz, 377; Guyton de Morveau, 382; Dr. Saunders, 388; Dr. Combe, 392; Dr. C. Wells, 395; A. G. Werner, 397; Dr. Birkbeck, 403; Dr. Bateman, 404.
Birkbeck, Dr. memoirs of 403
Birnie, Geo. on the yellow fever 144
Blackhall, Dr. on the redoubling pulse 164
Blackwall, on the water of the Thames at 171
Bladder, case of stone in the 36
——, state of it in reversed vagina 343
——, case where a wound in it terminated favourably 240, 346
——, calcareous matter found in that of a horse 348
Blake, Dr. Andrew, on abuses in medical practice from the influence of names 274
Bleeding, its benefit in fever 16
——, its inefficacy in phrenitis 104
——, efficacious in diseased action of the heart 130
——, method of practising it in the Tonga islands 201
Blegborough, Henry, on the treatment of croup 126
Blindness, alleged case of a young person who can read by the points of her fingers 81, 291, 366
——, scepticism expressed in that case 430
——, a person born blind restored to sight 518
Blistering, its inutility for mitigating internal inflammation 102
Blood, on the circulation of that of the arteries in wounds 11
——, effusion of it into the cellular texture of the lungs 54
——, its impetus diminished in debilitated vessels 163
Blue disease, remarks on the 117
——, case of it 152
Blumenbach, Professor, on the blue disease 117
Books, retrospective view of new medical 1
——, advice in the choice of 30
Books, catalogue of medical, 264, 352, 440, 522
——, new ones announced, 88, 175, 262, 436, 522
Boston in America, diseases prevalent there 165
Botany, excursions in search of plants round London 85, 349, 437
——, arrangement of plants in the midland counties of England 240
——, on the importance of vegetable geography 333
——, account of Humboldt's discoveries in ib.
——, experiments on the action of light on vegetables 381
Bougon, M. on a strangulation of the small intestines 169
Boyer, M. character of him as an anatomist 416
Braid, James, his account of a fatal accident in the mines of Leadhills 303
Brain, on the pathology of the 26
——, epilepsy caused by inflammation of the 43
——, case of induration of the 51
——, case of wounded 77
——, its influence on the heart 115
——, hydatids in the 258
——, case of rupture of the 464
——, on fever of the 487
——, on its morbid anatomy in typhus 491
Brandy, process of distilling it from potatoes 432
Bristol, statistical observations on that city and its neighbourhood 133
——, account of St. Peter's Hospital at 139
——, diseases of the poor at 141
Brodie, Mr. his experiments on the brain 115
Brown, Dr. J. on the system of 7
Bulam fever, its identity with the yellow fever 150
Burrows, Dr. on the laws relative to lunatic asylums 24
——, thanks of the Society of Apothecaries to 254
Bush, F. his suggestions for the extension of vaccination 107
Butter, description of rock 80
Buxton, Dr. his remarks on phthisis pulmonalis 97
Calcareous matter found in the bladders of horses 348
Caldwell, Dr. his new edition of Cullen's First Lines reviewed 155
Caledonian canal, on the current of the 172

Cancer, case of it in the right loin 344
———, observations on 478
Camphor, its rotatory motion on water 424
Cannon-balls, on the fatal effects of the wind of 265, 373
Carpue, Mr. his restoration of a lost nose 251
Carson, Dr. obscurity of his opinions on the motion of the arterial blood, 10; his observation on adhesive inflammation 11
Cases—of stone in the bladder, 36; injury to the head ending fatally after forty years had elapsed, 49; another after six years, 50; of diseases of the neck and inflammation of the trachea, 51; spasmodic affection of the larynx, 52; serous effusion of the lungs, 55; on diseases of the uterus, 58; spontaneous cure of an inguinal aneurism, 60; of shaking palsy, 74; of impregnation, 76; of a woman who wanted the left ovarium, 77; of croup, 126; of extraordinary periodical sickness, 129; of the swallowing of sulphuric acid, ib.; of lusus naturæ, 130; of vermis lumbricus in the intestinal canal, ib.; of diseased action of the heart, ib.; case of yellow fever, 146; of morbus cæruleus, 152; of hydrocephalus, 165, 166; of lithotomy, 168; of strangulation of the intestines, 169; history of an exophthalmia fungosa, with an engraving, 177; history of paralysis of the heart, 186; of phthisis pulmonalis, 191; of chronic diarrhœa, 193; of an aged person who cut new teeth at the end of life, 234; of diseased spine, 246; of catarrhus hepaticus, 249; of the re-union of the toes, 250; of a nose restored artificially, 251; of hydatid in the brain, 257; of hydrophobia by the bite of a cat, 258; of wounds by the wind of a cannon-ball, 266; of fatal aneurism after tying the internal iliac artery, 268; of placental presentation, 274; of obstinate constipation, 280; of an accident to the ear, 283; of disordered pupil by fits, 284; of a blind person, 291; of the death of Madame de Stael, 293; of elephantiasis, 298; of accidents in a mine, 303; of a fœtus with a double head and heart, 338; of cataracts, ib.; of a woman who lived without food eleven years, 341; of reversed vagina, 342; of cancer of the right loin, 344; of irregular conformation of the heart, ib.; of wounded bladder, 346; of calcareous matter in horses, 348; of morbus pediculosus, 350; of an osteo-steatomatous swelling, with an engraving, 355; farther particulars of extraordinary perception in a blind lady, 368, 430; of wounds in the stomach, 371; of knives swallowed, 372; of fractured skull by the wind of a ball, 373; of injury of the spinal cord, 406; of gun-shot wounds, 412; of lithotomy performed on a child, 420; extraordinary effects of cold in typhus, 433; of hydrophobia cured by alisma plantago, 435; of typhus, 437; of phthiriasis, 438; of puerperal fever cured by spirits of turpentine, 448; of rupture of the brain and its membranes in hydrocephalus internus, 464; of the efficacy of the nitro-muriatic acid, 466; of fractured ribs, 475; anus at the groin, 476; of a tumour of the face, 482; of stricture of the rectum, 488; of the discharge of part of the ileum, ib.; of tetanus, ib.; of colica stercorea, ib.; of femoral aneurism, 489; of diseased brain, 491; of an epidemic fever, 493; of injury to the nerves producing spasms, 504; of tetanus without a previous wound, 506; of her Royal Highness the Princess Charlotte, with remarks, 513; of poison from vipers, 517; of an enormous tumour on the head, ib.; of death by lightning, ib.; of a blind person restored to sight, 518.
Cat, hydrophobia occasioned by the bite of one 258
Cataract, observations on 23
———, on the operation of it by extraction 338
———, on the new method of extracting it 152
———, directions for the operation 420
Catarrhus hepaticus, cases of 249

Celsus, not an original author 227
Channing, Mr. his study of Arabic 230
Chapman, Edward, on the operation for cataract 152
Charlotte, Princess, her death, and remarks thereon 513
Charnock, Mr. remarks on his paper 38
Chaussier, M. his singular case of a woman who wanted the left ovarium 77
Chemistry has little connexion with medicine 1
———, on animal 2
———, amusements in 422
Cheselden, on the anatomical system of 414
Childers, observations on the yellow fever in the ship 146
Children, a particular species of ophthalmia to which weak ones are liable 185
Chinese, their operation of acupuncture 436
Chisholm, Dr. on the statistical pathology of Bristol and Clifton 133
Circumcision practised in the Tonga Islands 202
Clifton, statistical account of 133
———, diseases in the infirmary at 138
Cloquet, M. on a case of strangulation of the intestines 170
Clough, Mr. on the re-union of parts 250
Clutterbuck, Dr. his eulogium on Dr. Lettsom 125
———, his case of diseased action of the heart cured by bleeding 130
Colchicum autumnale, improved method of preparing it 76
———, fatal effects of it in gout 428
Cold, on the effects of it in inflammation 102
———, on the cold treatment of gout 285
———, extraordinary instance of its efficacy in typhus 43
———, another case of its benefit 489
Colic, benefit of quassia in 341
———, case of obstinate stercoraceous 488
Collectanea Medica, 39, 107, 198, 293, 377, 464
Colours, a blind person said to distinguish 291, 366, 430
Combe, Dr. Charles, memoirs of, 392; thanks of the court of the Lying-in Hospital to him, 394; his classical publications 395
Constipation, case of obstinate 280
Consumption, on the diagnosis of 32
———, remarks on the influence of regulated temperature in 97
———, effects of the weather in 176
———, analogy of chronic hepatitis, and 190
———, benefit of the digitalis in 339
Contagion, on the opinions of Dr. Bancroft on that subject 17
———, observations on infectious and contagious diseases 46
———, whether croup is contagious 128
———, promised dissertation on 143
———, proofs of the contagiousness of yellow fever 144
———, erroneous opinions on 160
———, proofs of it from the small-pox 161
———, remarkable conclusions on 345
———, account of fever arising from 495
Continental Medical Repertory, answer to an article in the 426
Cord, case of injury of the spinal 406
Cornwall, meeting of the Geological Society of 172
Correspondents, notices to, 88, 176, 264, 352, 440, 522
Corrosion, remarkable instance of it in iron works 431
Costiveness, case of obstinate 280
Cotton, corrosion of iron in the mill-work of 431
Craniotomist, description of a new forceps so called, with engravings 91
Crawford, Dr. on tonic medicines 25
Crichton, Dr. on hereditary diseases 110
Criminals, proposals for experiments on condemned 12
Croft, Sir Richard, his attendance on the Princess Charlotte 515
Croup, spasmodic affection of the larynx mistaken for 52
———, on the treatment of 126
———, observations on 128
Crowfoot, W. J. his experiments on arsenic 446
Croydon, botanical researches near 85
Cruikshank, Mr. his observations on the brain 115
Cullen, Dr. new edition of his First Lines, published in America 155

Cullen, Dr. vindication of his character 156
——, his definition of fever 158
——, account of his nosological system 212, 225
Cumberland, Mr. on the geology of Bristol and Clifton 143
Cupping recommended in erysipelatous inflammation 67
Curiosities, account of the discovery of some in natural history 35
——, on an optical natural one 291, 366
Curran, Mr. remarkable circumstance at his death 430, 516
Curry, Dr. on the physiology of muscular action 27
——, on the author of the theory concerning the secretion of pus 196
Curtis, J. H. account of his Treatise on the Ear, 326; his plagiarisms from Mr. Saunders 327
——, his vindication, 427; remarks in reply 428
Cutaneous diseases, observations on the classification of 19
Cuvier, M. his report of the Royal Academy of Sciences 333
——, his dissection of the Hottentot Venus 335
Daniell, John, his discovery of living animals in a solid rock 35
Darwin, Dr. observations on his medical opinions 6
——, animadversions on his nosology 215, 226
Davis, Dr. his invention of the craniotomist rewarded by the Society of Arts 77
——, description of that instrument 89
Davy, Sir Humphry, on useless words in science 18
——, Dr. his experiments on sea-water 76
—— on the nitro-muriatic acid 475
Deal, cases in the hospital at 66
Debility, on the causes of 6
Dee, observations on the water of the river 171
Dentition, observations on 234
Desgenettes, Dr. his Medical History of the Army of the East 445
Devonshire, Duchess of, her dissection secretly conducted 513
Diagnosis between chronic hepatitis and phthisis pulmonalis 32
——, contributions to 152
Diarrhœa, case of chronic 193
Digitalis, preparation of the tincture of 339
Dioscorides on a peculiar species of honey 204
Diseases, observations on infectious 46
——, on those of the neck 51
——, on hereditary 110
——, reports of, at Paris, 82, 259, 339, 340
——, reports of, in London, 83, 176, 264, 350, 438, 520
—— at Clifton, Gloucestershire 138
—— at Bristol 141
——, fatal one on board the Childers 146
——, on the classification of 157
—— prevalent at Boston 165
—— on those of the Tonga Islands 198
——, new arrangement of 210
——, their classes and orders 232, 306
——, on those of the spine 245
——, on cutaneous 309
——, on those of the ear 326
——, effects of acupuncture in chronic 436
——, virtue of nitro-muriatic acid in various 466
Dislocation, on the treatment of 61
——, case of that of the lower jaw 245
Dissections, 49, 50, 52, 55, 58, 76, 188, 195, 270, 359
Distension, on morbid excess of vascular 373
Distillation, on that of spirits from potatoes 432
Distortions, improved method of treating 516
Dog, the carotid of one obstructed by a cord 13
Donnell, John, on his trial for poisoning his mother-in-law 93, 95
Donnellan, Captain, evidence adduced in his case 95
Douglas, J. his case of wounded bladder 245, 346
Dropsy, efficacy of the tincture of digitalis in 339
Duncan, Dr. on preparations of bark 81
Dunn, John, his suggestions for the relief of the sick poor, and the improvement of the medical profession 511
Duodenum, on the action and diseases of the 26
Dutrochet, Dr. his cases of the re-union of parts 251
Dysentery, how to be treated 350
——, fatal cases of 438

Ear, on the physiology and diseases of the 326
——, explanation on the subject 428
Earle, Sir James, his death and character 348, 436
Earth, on that eaten by the Africans 79
East, on medical knowledge in the 204
Edgell, Richard, on turpentine in puerperal fever 447
Edinburgh Medical Essays, character of the 30
————, prize question of the Royal Medical Society of 79
————, extracts from the Medical and Surgical Journal, 133, 406, 487
————, proceedings of the Royal Society of 171
Edwards, G. N. his case of stricture of the rectum 488
Effusion, case of serous, into the lungs 55
Egg, singular formation of an 173
Egypt, on the state of medical and chemical knowledge in ancient 203
————, on the leprosy of 307
Electric fluid, subtilty of its action 4
Electricity, historic account of discoveries in 377
Elements, on those of organised substances 2
Elephantiasis, remarkable case of 21
————, observations on that disease at Madeira 301
———— the disorder of Job, 307, 461
————, Arabian accounts of it 309
————, its contagiousness questioned 311
————, further remarks on 461
Emden, Dr. his communication on a disease among children 185
————, on a paralysis of the heart 186
————, his case of an extraordinary tumour 355
————, on surgical operations 420
————, his vindication 426
————, his sketch of medicine in Russia 488
Epidemics, observations on 46
————, no two exactly alike 47
————, on the wintry one at Boston 165
Epilepsy, observations on 39, 126
————, fatal instances of 85
————, caused by worms 244
Ergot of rye, observations on 334
Eruptions, varieties of chronic 20
Erysipelatous inflammation, cupping recommended in 67
Essex, progress of vaccination in 174
Estlin, J. B. his case of diseased spine 245
Evacuations, on their utility in chronic diseases 436
Excitability, on the principle of 8
Excitement, on the Brunonian doctrine of 6
Exophthalmia fungosa, uncommon history of one successfully treated 179
Experiments, on making them on living animals 12
———— on arteries 68
———— on sea-water 76
———— on the detection of arsenic 92, 95
———— on the brain 115
———— on arsenic 172, 252
———— on the production of artificial ice 173
———— on ergot of rye 334
———— on the blind lady at Liverpool 366
———— in electricity 377
———— on vegetables 378, 380
———— on inflammable air 379
———— on the renovation of soils 381
———— chemical ones for amusement 422
———— effects of paste on iron 431
———— on the distillation of alcohol from the potato 432, 433
———— on arsenic 447
———— on nitro-muriatic acid 467
———— on the nerves 497
External world, on the connecting powers of the 9
Extracts, new method of preparing 431
Eye, on the diseases of the 23
——, on the new method of extracting the cataract 157
——, remarkable tumour on the 179
——, singular complaint in the eyes of children 185
——, treatment of inflammation of the eye among the people of Tonga 201
——, on the pupil, and accidents attending it 283
——, memoir on the operation for the cataract 338
——, further observations on that operation 420
——, a person born blind restored to sight 518
Face, remarkable history of tumour in the 483
Female organs, lusus naturæ in the 130

Fencing, accident occasioned by 77
Ferguson, Dr. on fever on 151
Fever, its ravages among the poor 83
——, remarkable one in the family of the Duke of Newcastle 84
——, on infectious 135
——, on typhus 137, 148
——, observations on marsh 139
——, on camp 143
——, objections to Cullen's definition of 158
——, on idiopathic 160
——, cases of typhus 165, 166
——, on inflammatory 176
——, on the nervous 264
——, benefit of cold in typhus 278
——, its frequency among the poor 350
——, cases of typhus 406
——, typhus cured by cold 433
——, imprudently conveyed into the country 437
——, epidemical in London 438
——, on turpentine in puerperal 447
——, benefit of nitro-muriatic acid in 467
——, on inflammation and brain 487
——, benefit of cold in typhus 489
——, morbid anatomy of the brain in typhus 491
—— prevalent in London 520
—— (yellow), on the contagious property of the 17
————, on the depletory treatment of 16, 18
——, on the spotted 48
——, instances of it in ships 144
——, on the Bulam 150
——, Dr. Ferguson on 151
——, on the mercurial treatment of yellow 487
Fibre, degrees of irritability in the animal 8
Filth, diseases occasioned by 140
Finger, restoration of a lost 250
Fire-bottle, experiments with the phosphoric 422
Fluids, experiment on the currents of opposite 423
Fœtus, observations on one without a head 7
————, another with a double head and heart 338
Forbes, John, his case of dislocation 245
Forceps, improvement in the construction of the 78
————, description of a new one 91
Fothergill, Dr. John, account of 121
Fourcroy, his part in the Encyclopedie 386
Fracas, account of a medical 430, 516
Fractures, method of treating them in the Tonga Islands 201
Fractures, case of fractured rib 475
————, advice in regard to 478
Frambæsia, or the yaws, observations on 221
France, progress of vaccination in 174
————, proceedings of the Academy of Sciences in 333
Franklin, Dr. anecdotes of 378
Friction successfully used in sprains 201
Functions, on the animal 9
Fungus hæmatodes, on the 481
Gaitskell, William, on croup 128
————, his case of lusus naturæ 150
Galen, his account of a peculiar kind of honey 204
Gallup, Dr. on contagion and infection 46
Galvanism, improvement in physiology from 9
Gangrene, strangulation of the intestines succeeded by 169
————, observations on the case 170
Garthshore, Dr. his biographical account of Dr. Ingenhousz 377
Geber, on the chemical works of 203
Generation, remarkable peculiarity in the female organs of 130
Geography, on vegetable 333
————, animals have their 334
Geological Society of Cornwall, proceedings of the 172
Gibbs, Dr. his oration before the College of Physicians 429
Gita, a disease in the Tonga islands, described 198
Glasgow, medical degrees conferred at 259
Glottis, on spasms of the 53
Glover, Rev. T. his case of a blind lady 366, 430
Golis, Dr. his case of a woman who died of paralysis of the heart 186
Good, J. M. observations on his System of Physiology 18
————, critical review of that work 210, 306
————, vindication of his system 360
————, the reviewer's reply to 453
Gordon, Dr. his case of injury in the spine 406
Gorget, objections to the use of the 169
Gout, improved method of preparing colchicum for the 76
——, on the application of cold in 285
——, review of a French treatise on the 331
——, fatal effects of colchicum in 428
——, new work announced on the 436
Gravesend, plants growing about 438

Greeks, on the medical knowledge of the 204
——, on the leprosy of the, 300, 302, 308
Gregory, Dr. his philosophical oration 78
Grieve, Dr. account of 121
Groin, instance of anus in the 476
Groningen, cases in the hospital of 420
Guilbert, Dr. review of his treatise on gout 331
Gum, tumour of the face springing from the 483
Gunshot wounds, on the treatment of 38
Hæmorrhage, on internal, after childbirth 462
Hall, Dr. his contributions to diagnosis 152
Haslam, Mr. his work on insanity commended 24
Hay, John, his cases of catarrhus hepaticus 249
Head, effects of injuries in the 49, 50
——, benefit of cold application to it in typhus 278, 489
——, a fœtus born with a double one 338
Heart, manner in which the blood enters the 10
——, influence of the brain on the action of the 115
——, on malconformation of the 117
——, case of diseased action of the 130
——, case of paralysis of the 186
——, on the suction influence of the 257
——, fœtus born with a double 338
——, irregular conformation of the 344
Hebb, Mr. his account of the resolutions of the Worcestershire Medical Society 79
Heberden, Dr. on epilepsy 42
—— on leprosy 311
—— on angina pectoris and asthma 320
Hedgehog, valuable qualities of the 431
Helling, Dr. his account of an enormous exophthalmic fungosa 179
Hendriksz, P. J. his account of surgical operations at Groningen 420
Hepatitis, analogy between phthisis pulmonalis and chronic 32
——, observations on that paper 190
——, benefit of the nitro-muriatic acid bath in chronic 470
Hernia, cases of 337
Herpes, on that of the ear 327
Higson, Mr. his vindication of Dr. Alderson's claim to a new method of treating gun-shot wounds 38
Hindus, their knowledge of syphilis 318
Hip-disease, observations on 60
Hoare, John, his account of natural curiosities 35
Hohnbaum, Dr. his account of a peculiar disease in the eyes of children 165, 185
——, his formulary of digitalis 339
Home, Sir Everard, on the formation of fat 57
——, on the passage of the ovum into the uterus 76
——, his improved method of preparing the colchicum 76
——, on injuries of the nerves 505
——, his case of tumour 517
Hooping-cough, its prevalence in America
Horner, Mr. his botanical work 333
Horses, calcareous matter found in the bladders of 348
Hottentot, account of a female 335
Howison, Dr. his case of morbus cœruleus 152
Howship, John, his practical observations in surgery and morbid anatomy 48
Humanitas, observations on the Worcestershire resolutions, by 165
——, answer to 289
Humboldt, M. his botanical researches 433
Hume, Mr. on his test for arsenic 244
Humerus, extraordinary disease in the 355
Hunter, John, on his doctrine of stimulus 14
——, on eruptions 20
——, change in medical language introduced by him 28
——, French report on the life of 77
——, his conduct on the trial of Donnellan 95
——, biographical account of 107
——, on his education 108
——, his classical knowledge 110
——, his improvements in medical science 114
——, his opinion on suppuration questioned 163, 196
——, on the teeth 238
——, on morbid poisons 315

Hutchinson, A. C. his practical observations in surgery reviewed 62
Hydatids, case of, in the head 258
Hydrocephalus, rupture of the brain in 464
Hydrophobia, remarkable case of 258
——, new remedy for the 435
Hydrothorax, on the diagnosis of 152
Hypochondriasis, account of a treatise on 24
Ice, improvement in the method of producing artificial 173
Iliac artery, on tying the internal 266
——, femoral aneurism cured by tying the external 489
Ileum, case of recovery after discharge of part of the 488
Infection, observations on 46
——, on fevers of 134
Inflammation, on that of the brain 45
——, case of that of the trachea 51
——, on the treatment of erysipelatous 66
——, effects of blistering and topical cold in 101
——, impetus of the blood diminished in 163
——, advantages of the cooling treatment of gouty and rheumatic 285
——, observations on spongoid 481
——, on brain fever and 487
Inguinal aneurism, case of 60
Ingenhousz, Dr. biographical memoir of 377
Insanity, remarks on the causes and treatment of 23
Insects, on the production of certain 47
Instruments, descriptions of new ones 91, 273, 348, 516
Intelligence, Medical and Philosophical, 76, 164, 249, 333, 426, 513
Intestines, the strangulation of the small 169, 170
Iron, extraordinary effect of paste on 431
Itch, observations on the 20
Jackson, Dr. his improved treatment of fevers 16
—— on the epidemic fever in America 165
Jaw, case of dislocation of the lower 245
Job, on the disease of 307, 461
Johnson, Mr. his improved method of preparing vegetable extracts 81, 431
Jones, Dr. his discovery of lymph in the inner coats of the arteries 14
——, observations on the experiments of 68
Kensington-gardens, plants in 349
Kerr, James, on poisoning with arsenic 92
Kerrison, Mr. thanks of the Society of Apothecaries to 256
King, R. H. his case of obstinate costiveness 280
Kinglake, Dr. on the analogy between chronic hepatitis and phthisis 32
——, reply to that paper 190
——, on the effects of blistering and topical cold 102
——, on the advantages of the cooling treatment of gout 285
——, on morbid excess of vascular distention 373
Language, change in medical 28
——, vindication of Dr. Cullen's 158
——, on a new classification of medical 210, 324
——, observations on 453
Larrey, Baron, objections to the priority of his claim in surgery 38
——, his case of an accident from fencing 77
Larynx, case of spasmodic affection of the 52
——, on diseases and wounds of the 474
Latin, on medical 31
Lazzaretto, Dr. his case of tetanus 488
Leander, report of the sick and wounded on board the 411
Laws, observations on those which affect medical practice 26
——, on those relating to apothecaries 259
——, on those relative to the poor 364
Leadhills, accident in the mine of 303
Lee, Dr. his description of elephantiasis 22
Lectures announced 78, 88, 260, 261, 262, 348, 440, 522
Leprosy, case of 23
——, observations on the 297, 301, 310
——, on that which afflicted Job 307
Leslie, Mr. improvement on his method of producing ice 173
Lettsom, Dr. account of 117
——, his case of vermes lumbricus 130
Leuce, observations on 312
Life, new theory of 29

Lightning, extraordinary instances of death by 517, 518
Lithotomy, accident in the operation of 168
————, method of operating in it at Groningen 420
Littleton, Nicholas, on the redoubling of the pulse 164
————, on the pupil and accidents attending it 283
Liver, on diseases of the 471
Liverpool, account of an extraordinary optical phenomenon at 81, 291, 366, 430, 519
————, bees found in a stone there 519
Lizards found in a chalk-bed 36
Lobenstein, Dr. on his mode of extirpating cataract 152
Locke, extract from his essay 30
Loin, case of cancer in the right 344
London, on the improvements in 84
————, transactions of the Medical Society of 126, 411
Lubbock, Dr. on the impetus of the blood 163
Lumbar abscesses, observations on 59
Lunatics, on the treatment of 23
Lungs, case of serous effusion in the 55
————, case of morbus cœruleus from ulceration of the 152
Lusus Naturæ, instance of 130
Lymph found in the coats of arteries 14
————, on coagulated 57
————, on the motion of 257
M'Evoy, Miss, the extraordinary case of 81, 291, 366, 430
M'Gregor, Sir James, on deaths by consumption 100
Madeira, on the leprosy of 298
Madness, on the causes of 23
Magnesia, efficacious in poison by arsenic 244
Male, Dr. on forensic medicine 25
Malta, on the climate of 99
Maniacs, on the treatment of 23
Marks, method of removing those of small-pox 208
Machel, Mr. account of his annular saw 348
————, his treatment of distortions 516
Marshall, John, on poisoning by arsenic 240
Martin, Dr. his account of medicine in the Tonga Islands 198
Mary-le-bone, population of the parish of 84
Matter, on the agency of inorganic 3
Mead, Dr. on nosological systems 224
Medical and Surgical Society of Worcester, resolutions of the 79
———— Society of Edinburgh, prize question of the ib.
———— topography, observations on 445
Medicine, historical retrospect of recent improvements in 1, 31
————, improvement more important in it than in surgery 16
————, on forensic 25
————, proceedings of the faculty of 77
————, defence of classical 155
————, practice of it in Tonga 198
————, state of it in the East 203
Medico-Chirurgical Transactions, account of the 411
Membranes, rupture of those of the brain 464
Mercurial treatment of yellow-fever, on the 487
Meteorological Journal kept in London 87, 175, 263, 351, 439, 521
———— reports at Paris 340
Midwifery, on the state of 16
————, improved instrument in 77
Mills, Dr. on the morbid anatomy of the brain in fever 491
Miln's history of the state of medicine in the East 203
Mines, fatal accident in the Lead-hills 303
Monro, Dr. Alex. account of his medical essays 30
————, account of his anatomy 418
Montaigre, Dr. on the disorders of Paris 340
Moors, the depositaries of knowledge 204
Morbid anatomy, observations in 48
———— effects of vascular distention 373
———— anatomy of the brain in fever 491
Morbus cæruleus, case of it occasioned by ulceration of the lungs 152
Morveau, memoir of Baron de 382
Mucus, white, in hooping-cough 165
Muscular action, on the physiology of 27
Nail, one swallowed and expelled 56
Names, on the influence of 274
Natural history, discoveries in 35
Navy, hints to practitioners in the 441
Neck, on some diseases of the 51
Necrosis, observations on 71
Negroes, their propensity to dirt-eating 79

Nervous system, view of its relations in health and disease 495
——, on diseases of the 503
——, on spasms of the 505
Newcastle, fever in the family of the Duke of 84
Newt, one found alive in a block of stone 35
Nitric acid, its antisyphilitic properties 429
Nitro-muriatic acid, its benefits in fevers 466, 480
Nomenclature, a new medical 232
Nose, process in the East of restoring a lost 251
Nosology, observations on its imperfection 19
——, account of a new system of 210, 306
——, defence of that system 360
——, reply of the reviewer 453
——, tables in 450
Odontia, definition and classification of 233
Oil, frictions with it used in sprains 201
Opium, administration of it in nervous disorders 508
Ophthalmia fungosa, case of 179
Ophthalmic disease, on a case of 185
Optical curiosity, particulars of an 291
——, another 518
Orders, series of classes and 232
Organic process, observations on the, 5
Osteo-steatomatous swelling, account of an extraordinary one, with an engraved representation 355
Ovum, on its passage from the ovarium into the uterus 76
Ovarium, one wanting in a woman who had borne eleven children 77
Palsy, observations on the shaking 24, 71
—— of the heart, remarkable case of 186
Paris, sittings of the faculty of medicine at 77
——, patients admitted in the hospital at 82, 259, 339
——, reigning maladies of 82, 340
——, medical cases at 169
——, meteorological reports at 259, 340
Parkinson, James, on the shaking palsy 24, 71
——, Dr. his nosological tables 449
Parr, Dr. on the medical dictionary of 227
Parry, Dr. on the principle of life 29
——, on his work on the nerves 507
Paste, its effects on iron 451
Percy, Baron, on wounds in the stomach 370
Peritoneal inflammation, on topical cold in 205
Peritonitis, benefit of turpentine in puerperal 447
Persian fire, an eastern term for syphilis 315
Philosophical society, anniversary of the 78
Phlogotica, or inflammations, definition of 317
Phosphoric fire-bottle, experiments with the 424
Phthiriasis, remarkable case of 350, 438
Phthisis pulmonalis, remarks on the influence of temperature on the 97
——, cases of, in London 176
——, analogy between chronic hepatitis and 190
Phrenitis, effects of topical cold in 103
Physicians, lectures at their college 26
——, picture of the Royal College of 403
——, annual meeting of 429
Physiology, on the uncertainty of 1
——, its application in the description and arrangement of diseases 240, 306
—— of the ear 326
——, vindication of a new system of 360
——, answer to that paper 453
Placenta, on internal hæmorrhage after expulsion of the 462
Plants, botanical description of British 240
Poison, on detecting that of arsenic 92, 95, 172, 244, 252, 446
——, characters of morbid 316
——, cases of that of the viper 517
Poor, resolutions of the Worcesshire Society respecting attendance on the 79
——, on the fever among the 83
——, answer to the resolutions concerning the parochial 164
——, on charges for attending the sick 289
——, society for relieving the ruptured 336
——, on insuring medical aid to the 364
——, suggestions for their relief 511
Population, on that of London 84
——, that of England 512
Portal, M. his account of the sickness and death of Mad. de Stael 293

Potass obtained from potato-tops 433
Potato, brandy extracted from the 432
———, potass obtained from the tops of the 433
Powell, Dr. his lectures on the brain 26
Powers, on the vital 7
Practitioners, on remunerating country ones for attending the poor 79, 164, 289, 364
———, hints to those in the navy and army 441
Pring, Mr. on the relation of the nervous system 495
Pritchard, Dr. on typhus fever 406
Prize question of the medical society 79
Puerperal peritonitis, benefit of turpentine in 447
Pulmonary complaints, on 54
Pulse, on the redoubling of the 164
Pupil, on accidents attending the 283
Purgatives, on the operations of 38
Purton, T. his arrangement of plants in the midland counties 240
Pus, on the secretion of 163, 196
Quarrier, Dr. his report of the state of the wounded in the action off Algiers 441
Quarantines, censure on 47
Quassia, benefit of 341
Quicksilver, its efficacy in colica stercorea 488
Rabies, epilepsy confounded with 45
Rectum, case of stricture of the 488
Reid, Dr. on nervous disorders 24
Renton, A. his case of recovery after discharging a portion of the ileum 488
Renwick, Dr. his extraordinary account of a blind lady 291
Retrospect of improvements in medicine and surgery 1, 31
Reunion of parts, cases of 250, 251
Rhazes, account of his treatise on the small-pox 206
Rheumatic inflammation, advantages of topical application of cold in 285
Rib, case of fractured 475
Roberts, Dr. on a case of elephantiasis 303
Robertson, Dr. Archibald, his case of femoral aneurism 488
Robertson, Dr. Wm. his advice to the practitioners in the army and navy 441
Roberton, Mr. his extraordinary conduct 516
Rock butter, description of 80
———, living animals found in the solid 35
Rolfe, W. D. his case of reunion by the first intention 250
Roux, M. on the operation for the cataract 338
Royal Society of London, meetings of the 76
——— of Edinburgh, papers of the 171
——— Academy of Sciences, proceedings of the 333
Royston, Mr. vindication of 489
Rupture of the brain, case of the 464
Ruptured poor, statement of the society for the relief of the 336
Rush, Dr. his treatment of Dr. Cullen censured 156
———, on the variety of disease 157
Russell, Dr. character of 121
Russia, state of medicine in 488
Rust, Dr. his description of an osteo-steatomatous swelling 355
Rye, on the ergot or spur of 334
Salernum, account of the university of 205
Salisbury, Mr. his botanical excursions 85, 349, 437
———, his lectures noticed 349
Saracens, the sciences indebted to them 205
Sarcocele, on the diagnosis and treatment of 421
Sarcoma, case of medullary 152
Saunders, Mr. plagiarisms from his work on the ear 326
———, Dr. Wm. memoirs of 388
Sauvages, on his nosological system 212
Saw, description of the annular 348
Scott, J. R. letter to him on the yellow-fever 144
———, Dr. H. on the nitro-muriatic acid bath 466
———, R. his defence of Mr. Royston 489
Scrotum, passage of the testicle into the 57
Sea, on the specific gravity of the 76
Sensorial power, on the 7
Serpent, description of a venomous one 335
——— eater, account of the 336
Sheppard, J. B. on the mercurial treatment of yellow-fever 487
Shot, observations on the wind of a 265, 373
Sicily, on the diseases of 97
Skin, effects of the atmosphere on the 79
Small-pox Hospital, report of the committee of the 81
———, on secondary cases of 106
———, abstract of an Arabian treatise on 206

Small-pox, how to destroy the marks of the 208
Society of arts, proceedings of the 78
———, proceedings of the geological 172
Sowerby, James, his account of plants in the midland counties 240
Spasms, objections to the term 53
———, on nervous 505
Spasmodic affection of the larynx, case of 52
Spinal cord, case of injury to the 406
Spine, case of diseased 245
———, case of fractured 475
Sprains, how treated in the Tonga Islands 201
Spurzheim, Dr. on the craniological doctrine of 23
Stael, Madame de, particulars of her death 293
Stahl, his zeal for the improvement of medicine 330
Stevenson, Mr. appointed oculist to the Duke of York 79
———, of Edinburgh, on the waters of the Dee 171
Stimulus, observations on 14
Stimson, Dr. his case of chronic diarrhœa 193
Stomach, on a disease of the 88
———, on wounds in the 370
Stone, case of, by the patient 36
———, unsuccessful operation for the 168
———, operation for it at Groningen 420
Stump, advice on the treatment of the bleeding 64
Strangulation of the intestines, fatal case of 169
Stricture of the rectum, case of 488
Sulphuric acid, case of the swallowing of 129
Suction influence, observations on that of the heart 11, 257
Suppuration, on the process of 163
Surgery, on the improvements of 12, 15
———, review of Howship's practical observation on 48
———, practical observations by Mr. Hutchinson 62
———, practice of it in the Tonga islands 198
Surgical operations in the hospital at Groningen 420
——— observations, account of those of Mr. C. Bell 476
Sutton, Dr. reply to him on consumption 97
Swallows, on the hybernation of 519
Swelling, an instance of uncommon osteo-steatomatous 355
Sydenham, character of 230
Sympathy, definition of the word 510
Syphilis, nosological description of 313
———, benefit of nitric acid in 429, 478
Teeth, observations on the natural history of the 233
Testicle, on its passage into the scrotum 57
———, enlarged, common at Tonga 200
———, on disorders of the 421
Tetanus, method of curing it in the Tonga islands 198
———, cases of 488, 506
———, on the general character of 507
Thames, observations on the water of the 171
Therapeutics, new elements of 330
Thillaye, M. his analysis of the life of Hunter 77
Tides, observations on the 171
Toad, one found alive in a bed of stone 35, 82
Tobacco, its benefit in tetanus 508
Toes, restoration of them after being separated 250
Tonga Islands, surgical practices in the 198
Tooke, Horne, anecdotes of 109
———, on etymologies 111
Topography, advice in regard to medical 443
Tourniquet, practical directions for the use of the 63
Trachea, cases of inflammation of the 51
———, case of a nail slipping into the 56
Truss Society, report of the London 336
Tumour, remarkable case of internal 153
———, uncommon, on the eye 179
———, extraordinary one of the face 483
———, monstrous one on the head 517
Turpentine, its benefit in puerperal peritonitis 447
Typhus, benefits of cold applications in 278
———, cases of it, with observations on the disease 406
——— cured by cold 433
———, on the morbid anatomy of the brain in 491
Uterus, on diseases of the 58
———, passage of the ovum into the 76
Vaccination, hint for the encouragement of 107
———, report of the national establishment on 174

Vaccination, its success in France 174
Vagina, malformation of the 130
———, case of it reversed 342
Valli, Dr. death of 345
Van Swieten, recommendation of his Commentaries 30
Varennes, M. his case of extraordinary abstinence 341
Vascular distension, morbid excess of 373
Vauquelin, M. on the spur of rye 334
Vegetable physics, discoveries in 333
Vegetables, chemical experiments on 381
————, new method of preparing extracts of 432
Vermis lumbricus, case of its perforating the intestinal canal and abdomen 132
Viper, description of the yellow 335
——, deaths by the poison of a 517
Virey, Dr. his account of remarkable worms in the human body 434
Vital principle, on the 29
Wandsworth, plants growing there 437
Want, Mr. his case of the fatal effects of colchicum 428
Ward, Mr. on the antisyphilitic properties of the nitric acid 429
Warner, Mr. his success in stopping the bleeding of the arteries 12
Water, on the rotatory motion of camphor in 424
Wells, Dr. memoirs of 395
Werner, Professor, memoir of 397
Wilkinson, Dr. his account of lizards found alive in a stone 36
Willan, Dr. observations on his system of classification 228
Wilson, Dr. A. P. on the opinions of 8
Wind, effects of that of a ball 265, 373
Wine, spirits of, distilled from potatoes 432
Wistar, Dr. account of his new system of anatomy 414
Woman, one who wanted the left ovarium 77
———— not more liable to consumption than man 99
Wood, Dr. on cold applications in typhus 278
————, on inflammation and brain fever 487
———, Joseph, on the hybernation of swallows 519
Worcester, resolutions of the Medical Society of 79
——————, animadversions on them 164
Worms, living one in the parietes of the abdomen 57
——— in the human body, account of 435
Wounds, on the spontaneous healing of 11
———, on those in the chest by balls 38
———, effects of one in the brain 77
———, how healed in the Tonga Islands 202
———, on those of the stomach 370
———, on the treatment of 477
Yeatman, J. C. on insuring medical aid to the poor 364
Yeats, Dr. his lectures on the pathology of the duodenum 26
Yellow fever, on the treatment of 17
—————, observations on the 144
—————, on the mercurial treatment of 487
Young, Dr. on the physiology of 216
—————, on the classification of fever 320
Zoology, observations in 334
Zoonologistical tables, outlines of 449
Zoonomia, observations on 8
Zugenbuhler, Dr. on the suction influence of the heart 257

### PLATES.

Dr. Davies's Craniotomy Forceps .......................... 91

Dr. Helling's Case of Exophthalmia Fungosa.............. 179

Mr. Atkinson's Instrument for tying the Internal Iliac Artery .................................................. 273

Dr. Rust's Description of an Osseous Swelling.............. 355

